# Experiences and beliefs related to exclusive breastfeeding and early supplementation in low income urban slums of Karachi, Pakistan- a qualitative study

**DOI:** 10.1186/s13006-025-00804-7

**Published:** 2026-01-08

**Authors:** Sajid Iqbal, Kheezran Ahmed, Maryam Mansoor, Ameer Muhammad, Sana Qaiser, Benazir Baloch, Yasmin Parpio, Yasir Shafiq, Muhammad Imran Nisar, Amy Sarah Ginsburg, Valerie Flaherman, Fyezah Jehan

**Affiliations:** 1 Shifa College of Nursing, Shifa Tameer-e-Millat University, Islamabad, Pakistan; 2 Department of Paediatrics & Child Health, Aga Khan University, Karachi, Sindh Pakistan; 3 Brigham and Women’s Hospital, Boston, MA United States of America; 4 Vital Pakistan Trust, Karachi, Sindh Pakistan; 5 Aga Khan University School of Nursing and Midwifery, Karachi, Pakistan; 6 Clinical Trials Unit, University of Washington, Seattle, WA United States of America; 7 UCSF Benioff Children’s Hospital, University of California San Francisco, San Francisco, CA United States of America

**Keywords:** Exclusive breastfeeding, Maternal and child health care, Feeding beliefs

## Abstract

In Pakistan, exclusive breastfeeding (EBF) is often difficult to establish and sustain in low-income urban settings due to limited access to antenatal and postnatal care, maternal undernutrition, socioeconomic constraints, sociocultural myths, and extended family influence. These factors frequently lead to early supplementation when breastfeeding is perceived as insufficient. We conducted a qualitative exploratory study using focus group discussions in four low-income urban slums of Karachi, Pakistan in January 2020. Our study aimed to explore the community beliefs and barriers relating to EBF practices. Nine focus group discussions were held with 84 caregivers, including mothers, fathers, and grandmothers of infants, as well as community health workers. Data was collected, translated and transcribed verbatim, and analyzed using an inductive thematic approach informed by a sociocultural conceptual framework. Overall, participants were aware of breastfeeding benefits, but many mothers lacked knowledge of proper breastfeeding techniques. The key dominant barriers we identified realting to sustained EBF included perceived inadequate milk production by mother, maternal health concerns, limited psychosocial support, and the influence of extended family members. In situations where breastfeeding was not possible, early complementary feeding was often preferred over expressed breast milk or formula. Caregivers were aware of the recommended feeding practices, yet adherence remained challenging as many mothers lacked practical knowledge of proper breastfeeding techniques and reported physical difficulties such as pain, perceived insufficient milk supple, and fatigue related to post partum workload. Concerns about infant size and growth frequently prompted early supplementation, often with liquids, herbal preparations, or semi-solid foods, rather than expressed breast milk or formula. While caregivers were broadly aware of recommended feeding practices, adherence remained difficult within the constraints of poverty, joint family systems, and limited health system engagement. Strengthening family-centered counseling which takes into account famuly dynamics during antenatal and postnatal care, empowering community health workers to address common myths, and improving support for mothers in low-resource households are essential strategies to improve EBF rates. Further research is also needed on the role of early supplementation in contexts of maternal undernutrition. This study aims to explore caregivers’ perceptions, practices, and challenges regarding exclusive breastfeeding and early supplementation in low-resource urban communities of Karachi, Pakistan.

**Supplementary Information:**

The online version contains supplementary material available at 10.1186/s13006-025-00804-7.

## Background

In Pakistan, exclusive breastfeeding (EBF) rates remain suboptimal, particularly in low-income urban settings. Despite global recommendations by the World Health Organization, EBF rates have stagnated, and pre-lacteal feeding persists [1, 2]. According to national surveys, less than half of infants are breastfed within the first hour of birth, and only a minority continue EBF for six months. Maternal factors such as poverty, illiteracy, limited antenatal and postnatal care, and household dynamics strongly influence feeding practices.

According to a 2018 National Nutritional Survey (NNS), less than half (45.8%) of Pakistani infants start breastfeeding within the first hour of birth [3].A 2021 study revealed that only 37.7% of women continue the practice of EBF [4]. Maternal factors such as poverty and illiteracy may influence attitudes towards EBF. About 55.8% of the mothers in the 2018 National Nutritional Survey were found to be illiterate and 46% belonged to the lowest wealth quintile [3]. In Pakistan, antenatal care quantity and quality remain suboptimal while postnatal care is rarely sought unless the mother or baby has an intercurrent illness [4]. These checkups can be important times where a mother can be given important knowledge about breastfeeding and give a chance for new mothers to discuss any challenges faced after birth. Pregnancy can be stressful and many mothers lack adequate psychosocial support for their mental health. Postnatally, maternal employment and lack of parental leave and lack of baby-friendly policies further influence eventual breastfeeding practices and lead to overburdened mothers [2, 5].

Pre-lacteal feeding is a risk factor for low adherence to EBF and other unhealthy practices such as early weaning. In Pakistan, cultural dogmas influence the high rates (up to 76%) of pre-lacteal feeding to newborns [6]. Early supplementation with commercial formula or readily available cow or goat milk is common. Widespread illiteracy and practices for example, locally held perceptions such as colostrum being considered harmful, or the belief that infants require supplementation to grow, continue to shape feeding practices in Pakistan [7]. Another culturally dominant factor affecting optimal breastfeeding in Pakistan is that the mother or even the immediate family is not the sole determinant. Many extended family members play a role in influencing the nutrition of a child which interferes with the EBF practice in Pakistan [8, 9].

In this milieu, it is crucial to unveil the barriers and facilitators of EBF and practices of early supplementation in the community to highlight specific tailored strategies and interventions that can be developed. This highlights the concern that closer scrutiny needs to be taken to look at the various cultural, socioeconomic and maternal factors leading to the decline in continuing EBF. Our study will provide insights into the perceptions and constraints related to breastfeeding and early supplementation in the low-resource setting of Karachi, Pakistan which are critical to designing family-centered interventions and strengthening community health worker counseling to address barriers to EBF in resource-constrained urban environments.

## Methods

### Study design and population

A qualitative exploratory study using focus group discussions was conducted in four urban settings; Rehri Goth, Ibrahim Hydri, Ali Akber Shah Colony and Bhains Colony of Karachi, Pakistan in January 2020. Nine focus group discussions (FGDs) were conducted with caregivers of infants younger than 12 months of age: seven FGDs with mothers, one FGD with fathers, and one FGD with mothers-in-law of the mothers, (paternal grandmothers). Participants in these communities typically belonged to lower socio-economic strata, with the majority of households relying on daily wage labor, fishing, or small-scale trading. Most families resided in congested housing with limited access to clean water and sanitation facilities. Literacy levels, especially among women, were low, consistent with national data showing over 50% maternal illiteracy in such settings. These socio-economic constraints shaped health-seeking behaviors and influenced feeding practices. The study was grounded in an interpretivist paradigm, recognizing that participants’ experiences and meanings are socially constructed within their sociocultural context.

The authors include eight women and four men with diverse professional experience, spanning various levels. The authors specialize in various areas of public health, pediatrics and child health and nursing and midwifery. This study has benefited greatly from the expertise, skills and field work, research and policy making of the authors.

### Data collection

Women were recruited from local clinics, convenience sampling method was used. After the identification of a potential participant, written informed consent was obtained in the local language. Most families lived in a joint family system where the parents and infants would live with other members of the family under the same roof. A total of nine FGDs were conducted until the point of saturation was reached, with one additional FGD conducted after saturation to confirm thematic completeness. All FGDs were moderated by a single female Research Associate with a Master of Arts in Sociology. The use of a single moderator ensured consistency in facilitation and probing across discussions; however, her training in sociology and position as an external researcher may have influenced participant responses and interpretation. To address this potential bias, reflexive practices were embedded throughout the study. The moderator maintained reflexive notes after each FGD, and regular team debriefings were held to reflect on assumptions, positionality, and emerging interpretations.

FGDs were audio-recorded, translated into English from local languages (Urdu and Sindhi), and transcribed for analysis. Translations were cross-checked by bilingual team members to ensure accuracy and preservation of cultural meanings. Reflexive discussions within the multidisciplinary research team were used to review coding decisions, challenge interpretations, and enhance analytic transparency.

### Ethics approval and consent to participate

This was a qualitative study, approval was sought from the Aga Khan University ERC (Ethical Review Committee) and the registrar of the Pakistani participating hospitals. ERC reference number: 2019-1434-5503.

In obtaining and documenting informed consent, the site investigators complied with local and domestic regulatory requirements and informed the participant that the study involves research, described the study’s risks, benefits, and alternatives and the confidentiality of records, provided contact information for answers to research questions as well as the voluntary nature of participation. Study staff also assessed participant understanding using open-ended questions to elicit any areas needing clarification. After the above procedures, informed consent was taken from willing participants by signature or thumbprint on the consent form. The consent forms included the purpose of the study, a description of the procedures to be followed and the risks and benefits of participation in a language they understand.

Participants in FGDs and interviews provided consent for themselves. Mothers provided consent for themselves and their children (newborn and, if applicable, the child prior to the newborn study baby) to be enrolled in Preventing Infant Malnutrition with Early Supplementation (PRIMES). Fathers provided consent for themselves when their data and measurements were collected.

The authors declare that the procedures were followed according to the regulations established by the Clinical Research and Ethics Committee and to the Standards for reporting qualitative research (SRQR) checklist [10]. All paper records, including signed informed consent, were kept in a locked office at the study site. No personal identifiers was transferred to UCSF. The de-identified measurements were stored securely in electronic form for a minimum of 10 years after the study.

### Theoretical framework

**Fig. 1:**
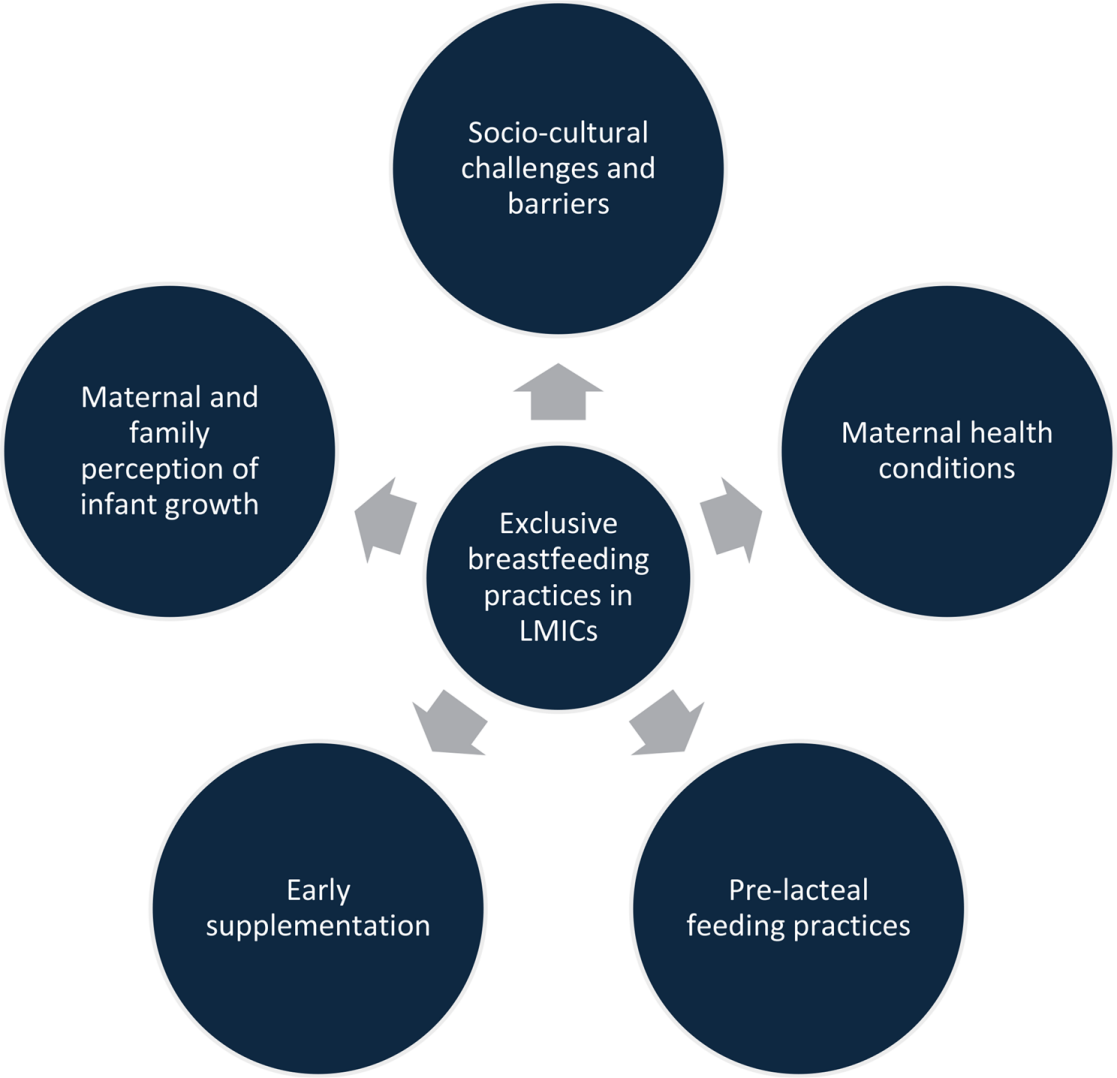
Theoretical framework

A theoretical framework (Fig. 1) was used for data collection and analysis to understand the sociocultural, household, maternal and infant-related factors that influence EBF and early supplementation. This framework was selected as it aligns with our study objectives of exploring multi-level influences on infant feeding, accommodating social, familial, maternal, and infant-level factors, and guiding both data collection and thematic categorization.

### Data analysis

Given the modest dataset, a manual inductive thematic approach was used for data analysis to allow closer engagement with the transcripts, guided by the thematic analysis framework described by Braun and Clarke. Similar codes were grouped together into more general concepts (i.e., subcategories) and further condensed into main categories. To ensure the validity of data interpretations, two investigators independently coded the transcripts. Discrepancies in coding were discussed in detail with a senior investigator, with reference to the original data, until agreement was reached. Throughout the analytic process, coding decisions and theme development were documented to maintain an audit trail and enhance transparency.

### Results

Findings are presented as themes that emerged from grouping similar codes into subthemes, which were then condensed into broader thematic categories, as outlined in the analytic process. A total of 84 caregiver participants (68 mothers, 8 fathers, and 8 mothers-in-law) were recruited (Table 1). Participants predominantly represented low socio-economic backgrounds. Among mothers (*n* = 68), more than half (55.8%) had no formal schooling and all were housewives. The majority were between 16 and 30 years of age, reflecting a young maternal population. Fathers (*n* = 8) were mostly engaged in fishing or daily wage labor, with 62.5% having no formal education. All mothers-in-law (*n* = 8) were illiterate and housewives. These socio-demographic characteristics are consistent with the broader context of urban slum populations in Karachi and shaped both knowledge and practices related to infant feeding. For example, limited maternal literacy and heavy reliance on informal employment among fathers often constrained access to health services and reinforced dependence on extended family advice The analysis of the FGDs identified several themes and subthemes related to caregivers’ perceptions, beliefs, and experiences regarding exclusive breastfeeding and early supplementation. The findings are organized into six main themes: (i) awareness about breastfeeding and its benefits, (ii) myths and misconceptions, (iii) challenges in breastfeeding, (iv) methods to improve breastfeeding, (v) support systems, and (vi) infant growth as an indicator of adequate feeding. In addition, community perceptions and practices regarding early supplementation, including challenges and acceptance at the community level, emerged as important cross-cutting themes. These themes are described in detail below using illustrative quotes from participants.

**Table 1:** Caregiver participant demographics

Caregiver	Number (Percent)
Mothers	
Age (years)	
16–20	16 (23.5%)
21–25	22 (32.3%)
26–30	20 (29.4%)
31–35	9 (13.2%)
35–40	1 (1.47%)
Education	
No schooling	38 (55.8%)
1–4	16 (23.5%)
5–9 years	1(1.47%)
10 + years	13(19.1%)
Occupation	
Housewife	68 (100%)
Marital Status	
Married	68 (100%)
Fathers	
Age (years)	
20–25	7 (87.55)
26–30	0
31–35	1 (12.5%)
Education	
No schooling	5 (62.5%)
1–4 years	0
10 + years	3 (37.5%)
Occupation	
Fisherman	5 (62.5%)
Labor	1(12.5%)
Teacher	1(12.5%)
Private job	1(12.5%)
Mothers-in-law (paternal grandmothers)	
Age (years)	
40–45	2 (25%)
46–50	4 (50%)
51–55	2(25%)
Education	
Primary	0
Secondary	0
Matric	0
Intermediate	0
Graduate	0
Illiterate	8 (100%)
Occupation	
Housewife	8(100%)

### Community perceptions, attitudes, and practices with breastfeeding

(i) Awareness about breastfeeding and its benefits

In 7 out of 9 FGDs, participants showed sufficient understanding of breastfeeding and its advantages. Many mothers shared that they exclusively breastfed their babies for six months as they were instructed by elders and health workers. One of the mothers explained, “People say that the child does not get ill with breast milk; he saves from the cold and the chest does not get disturbed as breast milk is good for the child.” (FGD-RG-R-1).

Most of the mothers were aware that breastfeeding protects the child from diseases. Mothers-in-law also had similar perceptions as they viewed breastfeeding as “nature’s gift” because it keeps the child healthy whereas fathers, while supportive, emphasized its economic benefit, considering breast milk healthier and more affordable than formula.

Many participants knew that the duration of breastfeeding should be six months. As one of the mothers stated, “We do not give even water to our children until six months.” (FGD-RG-R-1).

(ii) Myths and misconceptions

A few mothers-in-law discussed that babies do not have breast milk sometimes due to evil eye and that prayers helped solve this problem. “Once we feed the baby mother’s milk and she did not take it due to evil eyes. Then we took her to *molvi* (religious authority). He recited *dua* (prayer) upon her [and] then the baby started to feed.” (FGD-RG-R-5). These beliefs stem from long-standing cultural and spiritual explanations for infant feeding problems. Turning to religious authorities reflects the strong influence of faith and elders in family decision-making. Another myth amongst the mothers-in-law was that expressing breast milk is harmful for the baby. (FGD-RG, R-3). This view likely comes from limited guidance from health professionals. Without clear advice, such beliefs are passed down through generations and lead to avoiding expressed breast milk, even when direct breastfeeding is challenging.

(iii) Challenges in breastfeeding

#### Physical and emotional barriers

A few mothers reported having no difficulty while breastfeeding. However, in 6 of the 7 FGDs with mothers, women shared about their inability to breastfeed properly or indicated having insufficient breast milk. As one of the mothers revealed, “I was not able to sit because I had stitches. I had pain, and it was difficult to breastfeed for 5–6 days. While breastfeeding, the baby clenched his teeth like he is having episodes of fits” (R-12, Mother, pg12).

Mothers-in-law also reported concerns about insufficient milk. One stated, “Our baby cries too much because she does not have sufficient milk. My daughter-in-law had more milk at the time of the first child, but now in the second child, it is not enough. All people say to give him formula milk” (FGD, RG, R-6). Some older mothers compared current experiences with their past, noting that insufficient milk was not a problem in earlier generations.

Other physical challenges included babies vomiting after breastfeeding. Some mothers-in-law associated continuous breastfeeding with maternal weakness and the inability to bear or feed another child.

Many primigravid mothers also lacked awareness of proper breastfeeding techniques, which added to difficulties. One participant explained, “Initially, child was breastfeeding [and] then [the mother] had bleeding from breast [and] had to clean the breast first [and] then feed breast milk. The child is feeding breast milk but vomits out” (FGD, R-9).

Household responsibilities further complicated breastfeeding. Mothers noted, “When we breastfeed the child, husband or mother-in-law calls me for work [and] then it gets difficult. We get irritated and find it difficult to breastfeed the child” (FGD, R-9).

#### Role of health professionals and family guidance

Some mothers were advised by doctors to express milk. Family members also guided mothers to feed babies with a spoon. One mother reported, “As she (baby) never feed my breast milk, my breast milk was spoiled then I started to express in the feeder. I was not able to feed through my breast, they were ulcerated then she fed in the feeder for 5–6 days, that’s why she stopped feeding breast milk. I used to express and give her in the feeder then she had a habit of the feeder. I expressed in the feeder. I also had a wound, it was bleeding because of this reason I used to express through a pump and give in the feeder” (FGD, R-6).

In some cases, infants were too weak to feed immediately after birth. One mother shared, “In the initial days, the child is too small and has to struggle after the delivery, so the child does not feed. The difficulty is that the child is weak when born so he does not feed because of weakness” (FGD, RG, R-6).

Occasionally, cross-nursing by other female family members was reported. While rare, this practice is culturally accepted in some households and provides temporary support when mothers face difficulties. Ethical and cultural norms emphasize consent, family approval, and hygiene during such practices, reflecting communal responsibility for infant care in South Asian contexts [11]. One mother explained, “I did it once. I went out of the house to my aunty and she was at home and crying, so my sister-in-law feeds her breast milk” (FGD, RG, R-2). Fathers noted that breastfeeding challenges were sometimes linked to maternal health issues, such as backaches, dizziness, or breast abscesses, and also mentioned that babies vomited if they had chest congestion or weakness.

(iv) Methods to improve breastfeeding

Many of the mothers knew that drinking enough water improves breastfeeding. Mothers also believed that consuming cumin, dates, popcorn, apples, and milk improves breastfeeding and reported that older women advised them to eat lentils in liquid form. Mothers reported seeking advice from nurses and adopting strategies such as drinking fluids or frequent breastfeeding. Grandmothers often emphasized diet and traditional remedies, while some fathers stressed the role of doctors or health workers rather than family advice. One mother stated,

“The mother should eat more so she has proper milk supply for the child. I have more healthy food so my feed is sufficient. My doctor told me to eat 7 dates with milk and an apple in a day.” (FGD-RG, R-2).

Mothers-in-law knew that sufficient milk supply depends on the mother’s diet. They also encouraged spiritual support like prayers or taking the child to a religious authority (*molvi*) to improve breastfeeding. Mothers also reported expressing breast milk using a pump when they have sufficient milk supply to avoid chest pain.

“When I had excessive milk, then I had a lot of problems with breastfeeding. Someone suggested expressing from the pump, since I was not able to breastfeed properly and I had pain in my chest. Then I expressed from the pump and fed the baby from a feeder or spoon” (FGD-RG, R-10).

(v) Support system

In 5 FGDs, mothers reported receiving support from women in their families, health workers, and health professionals to help with exclusive breastfeeding. One participant explained, “Health workers come to check and observe breastfeeding. They advise drinking water and having good food” (FGD-R-3). Support from female family members, such as mothers, sisters, or mothers-in-law, was often hands-on, including holding the baby, assisting with latching, and providing practical guidance. One mother described, “My sister and mother helped me. I soaked the milk in cotton and then fed my breast milk. She picked up and held my child then attached with me. Child was not gripping the breast properly. He used to loosen the grip, so she held the child in lap” (R-18, Mother).

Fathers, in contrast, played a more passive role. They generally did not intervene directly in breastfeeding practices and left decisions to the mothers. Mothers-in-law often had a supervisory or advisory role, influencing feeding practices and encouraging traditional methods, while fathers primarily provided indirect support, such as permitting the mother to follow guidance from elders or health workers. These gendered patterns highlight how practical, hands-on assistance is largely provided by female relatives, whereas male family members offer emotional or logistical support without direct involvement in breastfeeding.

(vi) Infant growth as an indicator

In 6 FGDs, mothers expressed worry about the size and weight of their baby. Few mothers think that their breast milk does not give strength or suit the child. While some mothers-in-law were not worried about the baby’s height and weight, others were concerned about the babies not growing:

“My daughter-in-law is stressed about her daughter. She is like a dwarf; all people laugh at her.” (FGD-RG-R-4).

These perceptions of growth directly influenced feeding decisions. Mothers who worried about insufficient growth often consulted doctors before introducing food or liquids and considered more frequent feeding or night feeds to compensate for perceived deficiencies in breast milk. Fathers were less engaged in these decisions, often deferring responsibility to female relatives, while grandmothers relied on home remedies or traditional practices such as massage and sunlight exposure. Mothers-in-law described additional strategies for addressing insufficient growth, including massaging the child with olive oil, exposing the baby to sunlight, using spiritual methods, and maintaining hygiene.

### Community perceptions about early supplementation

#### Belief’s and perceptions

Across the 9 FGDs, participants expressed mixed views about early supplementation. Grandmothers and some mothers emphasized the risks of giving other foods or liquids before six months, often associating formula or early complementary feeding with indigestion, diarrhea, or vomiting. One mother-in-law stated, “Nowadays it is fashion that the daughter-in-law is going to a wedding, then she keeps feeder along with her so that will not have to give breast milk over there.” (FGD, RG, R-2) Another said, “I say that the baby’s chest will be disturbed from formula milk. What comes from the power of Allah is different.” (FGD, RG, R-3).

Many mothers-in-law were against early supplementation before six months, while others acknowledged that certain liquids or herbal tonics were unavoidable. Fathers generally expressed conditional acceptance if breast milk was perceived as insufficient. These mixed messages illustrate internal contradictions within households, where elders may both discourage and, at times, promote early supplementation depending on context.

Community norms also favored complementary feeding after six months, reflecting the belief that introducing food too early could cause illness or weakness. Mothers often sought guidance from both family elders and health professionals. One mother reported, “Yes, I discussed so they say to give complementary food like porridge, khichri, and custard so I gave these things.” (FGD-RG, R-9).

#### Practices related to early supplementation

Despite beliefs, practices varied. Some mothers occasionally used formula, “ghutti” (herbal tonics), or porridge when they were away from home, while continuing breastfeeding when present. Others avoided early supplementation entirely, believing breast milk was sufficient. A few fathers deferred feeding decisions to female relatives, and grandmothers strongly influenced the actual feeding practices.

Early supplementation also posed practical challenges. Mothers highlighted the time and effort required for bottle feeding, including washing and sterilizing bottles, preparing formula, and storing it safely. These hygiene and workload concerns were particularly burdensome in low-resource households, further shaping decisions to prioritize breastfeeding when feasible.

Some mothers-in-law suggested gradual transitions to supplementation, such as avoiding night feeds, feeding slowly, and burping the baby after feeds, to minimize problems while maintaining breastfeeding.

## Discussion

Community experiences regarding EBF and early supplementation varied among participants. Our study highlighted the various drivers and barriers to these practices. Our findings align with the conceptual framework employed in this study. At the sociocultural level, myths surrounding colostrum and the influence of elders significantly impacted feeding practices. At the household level, joint family dynamics often led to a shift in decision-making power away from mothers. At the maternal level, poor nutritional status, inadequate psychosocial support, and postpartum workload emerged as major barriers to exclusive breastfeeding. Finally, at the infant level, perceived weakness or inadequate growth prompted early supplementation. This alignment underscores the interplay of structural and interpersonal factors shaping breastfeeding practices in urban slums of Karachi.

Although our findings suggest that overall knowledge about the benefits of breast milk was sufficient, many mothers were not aware of the importance of exclusive breastfeeding. These findings concur with previous studies which highlight the lack of awareness regarding EBF [3, 12].

In certain sociocultural settings, older members play a pivotal influence on influencing the child’s breastfeeding and supplementation decisions. Our findings concluded that parents in the community actively consulted and followed their elders’ opinions regarding feeding practices. While in some scenarios, the support proved beneficial such as when an older relative helped the mother when it was time to breastfeed the baby or provided emotional support. However, in other scenarios, the advice of elders had a negative impact. Many elders advised discarding colostrum and expressed breast milk as they believed it contained germs, which undermined mothers’ ability to breastfeed effectively. Family influence persists despite health education interventions because cultural norms and intergenerational knowledge often outweigh formal health messaging. Awareness campaigns in Pakistan largely target mothers alone. Although these interventions improved maternal knowledge, they did not significantly change overall breastfeeding practices [8, 9]. This highlights the need for integrated family-focused campaigns that engage fathers and elderly family members. Moreover, breastfeeding interventions should be embedded in routine antenatal and postnatal care, as these encounters provide critical opportunities for practical guidance and support [8, 9].

Our study also showed that pre lacteal feeding was common. Pre-lacteal and inadequate complementary feeding practices are recognized predecessors that can lead to lactation failure. Similar trends of such practices are apparent in other regions in South Asia, such as in India [13] where maternal knowledge gaps, sociocultural beliefs, and extended family influence were reported as key determinants of breastfeeding practices [14–16]. These results reinforce the need for family-inclusive community education and support interventions tailored to local contexts.

These findings highlight the importance of promoting educational programs and counseling by healthcare workers with an emphasis on women belonging to lower socioeconomic classes.

Most mothers showed a negative response towards early supplementation. The use of formula milk was discouraged, and early supplementation was done concerning liquids (honey, water) or herbal tonics. Proper emphasis should be given to discouraging early supplementation, and counseling regarding complementary breastfeeding after 6 months of age should be encouraged to prevent future stunting, wasting, and nutrient deficiencies [17].

Maternal health was identified as key barrier influencing feeding practices. Maternal undernutrition and subsequent inadequate milk production lead to undermined maternal confidence and early cessation of breastfeeding [18, 19]. Lactation support through nurses and mental health care support can prove to be an empathetic and reasonable solution to such problems [20, 21].

Mothers in our study also perceived breast abscesses and related complications as resulting from overfeeding, improper attachment, or even “spoiled” expressed milk. Within our conceptual framework, these perceptions fall under maternal-level barriers, where misinformation and lack of support contribute to breastfeeding challenges. Preventive measures such as early guidance on correct latch, management of engorgement, and timely medical care are essential to dispel myths and reduce unnecessary cessation of breastfeeding [22, 23].

Taking care of a child, especially after birth where a mother must take care of not just herself and the child but also the responsibilities at home can be a challenging task and can prove to be a mentally and physically strenuous time for her. In our study mothers talked about the importance of emotional support either from health care workers or even members of the family as a positive factor. There are several provincial and national policies that encourage breastfeeding in Pakistan. Pakistan’s Infant Young Child Feed strategy (2016–2020) highlighted actions needed to improve feeding practices and nutrition of infants and young children as well as increasing rates of early initiation of EBF and continued breastfeeding [24]. More recently, the Pakistan Maternal Nutrition Strategy 2022–2027 has emphasized postpartum maternal nutrition and integrated breastfeeding support within broader nutrition and health service delivery priorities, further anchoring breastfeeding promotion in national health policy [25]. Although Pakistan has a Maternity Leave Law, women who work in the informal sector do not have access to entitlements such as paid maternity leaves [20]. The absence of lactation support, mental health care, and lack of maternity leave in an informal sector can lead to deteriorating breastfeeding rates and long-term detrimental effects on the mother and child [23].

### Strengths and limitations

This study has several limitations that warrant consideration for future research on this topic. Firstly, as a qualitative study conducted in urban slums of Karachi, its findings may not be generalizable to all settings. Secondly, focus group discussions (FGDs) relied on self-reported practices, which may have introduced recall or social desirability bias, particularly for sensitive behaviors such as pre-lacteal feeding. The use of convenience sampling may have excluded mothers who were harder to reach or less health-conscious. Thirdly, the process of translation from local languages (Urdu and Sindhi) into English may have introduced translator bias, potentially affecting the nuance and cultural meanings of participant responses. Additionally, the positionality of the moderator, who was an external female researcher with a sociology background, could have influenced participant responses despite efforts at reflexivity. Despite these limitations, the study provides valuable contextual insights into the barriers and facilitators of exclusive breastfeeding in marginalized urban populations.

## Conclusions

In Pakistan a more nuanced understanding of the experiences and beliefs of EBF and early supplementation is needed. Although care givers were aware about the benefits of breastfeeding to the mother and infant their understandings of the experiences and beliefs regarding EBF and early supplementation demonstrated several gaps which could be addressed through inclusive integrated family centric campaigns (including pivotal extended family members). Adequate support should be provided to mothers during antenatal and postnatal periods, with community health workers playing a stronger role in addressing myths, offering psychosocial support, and guiding practical feeding techniques through routine home visits and structured counseling sessions.

Perceptions regarding the role of early supplementation in promoting infant growth remain unclear and require further research. At the same time, breastfeeding interventions and policies should be scaled up and implemented to improve EBF rates, taking into account sociocultural beliefs, maternal challenges, and systemic barriers. Safe and less costly alternatives for mothers unable to access commercial formula—such as fortified local complementary foods, peer counseling, and community-based lactation support—should also be explored and integrated into programs.

## Supplementary Information

Below is the link to the electronic supplementary material.

Supplementary Material 1

Supplementary Material 2

Supplementary Material 3

## Data Availability

The authors confirm that the data supporting the findings of this study are available within the article and its supplementary material. Raw data that support the findings of the study are available from the corresponding author, upon resonable request.
