# Supplementary material for: Experiences and beliefs related to exclusive breastfeeding and early supplementation in low income urban slums of Karachi, Pakistan- a qualitative study
Source: Int Breastfeed J. 2026 Jan 8;21:15. doi: 10.1186/s13006-025-00804-7 (PMC12869958; doi:10.1186/s13006-025-00804-7)
Supplement: Supplementary file 1 — Supplementary Material 1 [file 13006_2025_804_MOESM1_ESM.docx]

**INFORMED CONSENT TO PARTICIPATE IN RESEARCH**

**Study Title:** *Primes 1: Preventing Infant Malnutrition with Early Supplementation*

**Introduction:**

This is a research study about newborn weight and breastfeeding and what you think about these

things. The study research staff will explain this study to you.

Research studies include only people who choose to take part. Please take your time to make

your own decision about participating, and discuss your decision with your family or friends if

you wish. If you have any questions, you may ask the study staff.

You are being asked to take part in this study because you are a parent of an infant that is less

than 12 months old or you are a healthcare worker in this area.

**Why is this study being done?**

The purpose of this study is to understand your thoughts and feelings about breastfeeding, other

ways of feeding infants, and infant weight changes.

**How many people will take part in this study?**

About 30-72 individuals will take part in this study per site. There are 3 parts to the study, and

you are being asked to participate in one of these parts. Some people are being asked to

participate in an interview, and some people are being asked to participate in a focus group. If

you are a parent or a healthcare worker, you are being asked to participate in a focus group with

your peers. If you are a pediatrician or a member of the international aid community, you are

being asked to participate in an interview.

**What will happen if I take part in this research study?**

If you agree, the following procedures will occur:

If you are a parent or a healthcare worker, you will participate in a focus group discussion with

approximately 6-12 of your peers. The focus group discussions will last about 90 minutes.

During that time, you will be able to participate in a discussion about breastfeeding, formula,

and infant weight.

If you are a pediatrician or a member of the international aid community, you will participate in

a semi-structured interview with a member of our research team. The interview will last

approximately 60 minutes.

**Focus Group:**

• The focus group discussion will be audio-recorded. After the focus group discussion, a

member of the research team will listen to the recording, write down everything that was

recorded, and remove any personal identifiers. This process is called transcription.

● You and your peers will be given prompts, such as “what is the best thing about

breastfeeding” and you will have a chance to talk about the topic.

● You will be asked to share your thoughts about topics like breastfeeding, formula, and

infant weight.

**Interview:**

● The interview will be audio-recorded. After the interview, a member of the research

team will transcribe the recording and remove any personal identifiers.

● You will meet one-on-one with a member of the research team.

● You will be asked questions about your thoughts on breastfeeding, formula, and infant

weight.

**How long will I be in the study?**

If you are in a focus group discussion, the study will take a total of about 90 minutes. If you take

part in an interview, the study will take a total of about 60 minutes.

**Can I stop being in the study?**

Yes. You can decide to stop at any time. Just tell the study researcher or staff person right away if you wish to stop being in the study.

Also, the study researcher may stop you from taking part in this study at any time if he or she

believes it is in your best interest, if you do not follow the study rules, or if the study is stopped.

**What side effects or risks can I expect from being in the study?**

There are very few risks anticipated from this study. It is possible you may feel uncomfortable

discussing breastfeeding or formula use. During the focus group discussion, some of the

discussion topics or opinions from your peers may make you uncomfortable.

**Are there benefits to taking part in the study?**

There will be no direct benefit to you from participating in this study. However, the information

that you provide may help health professionals better understand or learn more about infant weight

changes, infant feeding patterns, and what people think about feeding infants.

**What other choices do I have if I do not take part in this study?**

You are free to choose not to participate in the study. If you decide not to take part in this study,

there will be no penalty to you. You will not lose any of your regular benefits, and you can still

get your care from our institution the way you usually do.

**Will information about me be kept private?**

We will do our best to make sure that the personal information gathered for this study is kept

private. However, we cannot guarantee total privacy. Your personal information may be given

out if required by law. If information from this study is published or presented at scientific

meetings, your name and other personal information will not be used.

Authorized representatives may review your research data for the purpose of monitoring or

managing the conduct of this study.

The researchers will ask you and the other people in the group to use only first names during the

group session and during the interview. They will also ask you not to tell anyone outside the

group what any particular person said in the group. However, the researchers cannot guarantee

that everyone will keep the discussions private.

**Are there any costs to me for taking part in this study?**

No.

**Will I be paid for taking part in this study?**

You will not be paid for taking part in this study.

**What are my rights if I take part in this study?**

Taking part in this study is your choice. You may choose either to take part or not to take part in

the study. If you decide to take part in this study, you may leave the study at any time. No

matter what decision you make, there will be no penalty to you in any way. You will not lose

any of your regular benefits, and you can still get your care from our institution the way you

usually do.

**Who can answer my questions about the study?**

You can talk to the researcher(s) about any questions, concerns, or complaints you have about

this study.

If you wish to ask questions about the study or your rights as a research participant to someone

other than the researchers or if you wish to voice any problems or concerns you may have about

the study, please call the Ethical Review Committee (ERC).

**CONSENT:**

You have been given a copy of this consent form to keep.

PARTICIPATION IN RESEARCH IS VOLUNTARY. You have the right to decline to be in this study or to withdraw from it at any point without penalty or loss of benefits to which you are otherwise entitled.

If you wish to participate in this study, you should sign below.

______________ ______________________________________

Date Participant's Signature for Consent

______________ ______________________________________

Date Person Obtaining Consent

______________ ______________________________________

Date Name and signature of witness
