# Supplementary material for: Experiences and beliefs related to exclusive breastfeeding and early supplementation in low income urban slums of Karachi, Pakistan- a qualitative study
Source: Int Breastfeed J. 2026 Jan 8;21:15. doi: 10.1186/s13006-025-00804-7 (PMC12869958; doi:10.1186/s13006-025-00804-7)
Supplement: Supplementary file 2 — Supplementary Material 2 [file 13006_2025_804_MOESM2_ESM.pdf]

آغا خان یونیورسٹی
THE AGA KHAN UNIVERSITY

09-Jan-2020

Dr. Fyezah Jehan
Department of Paediatrics and Child Health
The Aga Khan University
Karachi

Dear Dr. Fyezah Jehan,

Re: 2020-1434-7169. Fyezah Jehan: PRIMES (Preventing Infant Malnutrition with Early Supplementation)

Thank you for your submission.

The proposed amendments have been reviewed and approved. Please note that this only constitutes an approval to your requested amendment(s) till the current validity period of your ERC approval. If any further extension is required, then kindly apply for an extension separately by creating a subform.

List of amended document(s) approved with this submission.

Submission Document Name	Submission Document Date	Submission Document Version
PRIMES Pakistan Aim 2 Data Collection Instrument (Version 3.1)	12-Dec-2019	3.1
PRIMES Pakistan Aim 2 Data Collection Instrument(URDU) - (version 1.1)	12-Dec-2019	1.1
PRIMES Pakistan Qualitative Guides Aim 1(URDU)_version 1.1_29-11-2019	29-Nov-2019	1.1
PRIMES Pakistan Site-specific Protocol (Version 1.2)	21-Dec-2019	1.2

Any changes in the protocol or extension in the period of study should be notified to the committee for prior approval. All informed consents should be retained for future reference.

Thank you.

Sincerely,

Dr. Rehana Siddiqui

Chairperson

Ethics Review Committee
